# Regular Physical Exercise Modulates Iron Homeostasis in the 5xFAD Mouse Model of Alzheimer’s Disease

**DOI:** 10.3390/ijms22168715

**Published:** 2021-08-13

**Authors:** Irina Belaya, Nina Kucháriková, Veronika Górová, Kai Kysenius, Dominic J. Hare, Peter J. Crouch, Tarja Malm, Mustafa Atalay, Anthony R. White, Jeffrey R. Liddell, Katja M. Kanninen

**Affiliations:** 1 A.I. Virtanen Institute for Molecular Sciences, University of Eastern Finland, 70211 Kuopio, Finland; irina.belaia@uef.fi (I.B.); nina.kucharikova@uef.fi (N.K.); veronika.gorova@uef.fi (V.G.); tarja.malm@uef.fi (T.M.); 2 Department of Biochemistry and Pharmacology, The University of Melbourne, Melbourne, VIC 3010, Australia; kai.kysenius@unimelb.edu.au (K.K.); pjcrouch@unimelb.edu.au (P.J.C.); jliddell@unimelb.edu.au (J.R.L.); 3 School of BioSciences, The University of Melbourne, Melbourne, VIC 3010, Australia; dominic.hare@uts.edu.au; 4 Atomic Medicine Initiative, University of Technology Sydney, Sydney, NSW 2007, Australia; 5 Institute of Biomedicine, University of Eastern Finland, 70211 Kuopio, Finland; mustafa.atalay@uef.fi; 6 Mental Health Program, QIMR Berghofer Medical Research Institute, Brisbane, QLD 4006, Australia; tony.white@qimrberghofer.edu.au

**Keywords:** Alzheimer’s disease, 5xFAD mouse, regular voluntary exercise, iron, hepcidin, il-6, cortex, skeletal muscle

## Abstract

Dysregulation of brain iron metabolism is one of the pathological features of aging and Alzheimer’s disease (AD), a neurodegenerative disease characterized by progressive memory loss and cognitive impairment. While physical inactivity is one of the risk factors for AD and regular exercise improves cognitive function and reduces pathology associated with AD, the underlying mechanisms remain unclear. The purpose of the study is to explore the effect of regular physical exercise on modulation of iron homeostasis in the brain and periphery of the 5xFAD mouse model of AD. By using inductively coupled plasma mass spectrometry and a variety of biochemical techniques, we measured total iron content and level of proteins essential in iron homeostasis in the brain and skeletal muscles of sedentary and exercised mice. Long-term voluntary running induced redistribution of iron resulted in altered iron metabolism and trafficking in the brain and increased iron content in skeletal muscle. Exercise reduced levels of cortical hepcidin, a key regulator of iron homeostasis, coupled with interleukin-6 (IL-6) decrease in cortex and plasma. We propose that regular exercise induces a reduction of hepcidin in the brain, possibly via the IL-6/STAT3/JAK1 pathway. These findings indicate that regular exercise modulates iron homeostasis in both wild-type and AD mice.

## 1. Introduction

Alzheimer’s disease (AD) is a chronic neurodegenerative disorder mainly affecting the aged population and characterized by progressive memory loss and cognitive impairment [1]. Although age-related and genetic risk factors are implicated in the majority of all AD cases, a large proportion of cases are linked with lifestyle factors such as physical inactivity, unhealthy diet, social isolation, and living in polluted locations [2]. Due to the lack of effective medical treatments, more efforts should be focused on modulating lifestyle factors such as physical activity in efforts to slow or prevent disease progression. Regular physical exercise has huge impacts on health, reducing risk of cardiovascular diseases, chronic metabolic diseases, psychiatric disorders, and dementia enhancing adaptation against stress, anti-inflammatory action, and normalization of metabolic status [3,4]. In the brain, physical exercise positively affects neuronal plasticity and memory, also reducing age-related declines in synaptic function and adult neurogenesis [5,6]. In skeletal muscle, the major organ responding to exercise, physical exercise increases metabolic activity, enhances antioxidant systems, and reduces inflammation [7].

Age-related diseases including AD are associated with dysregulation of biometal homeostasis, which is implicated in disruption of critical cellular processes [8,9,10]. For example, excess of redox-active iron (Fe^2+^) or dysregulation of iron metabolism can generate intensively reactive oxygen species (ROS) and lead to oxidative stress, disruption of redox-homeostasis [11,12], and ferroptosis [13]. In AD, dysregulation of iron metabolism and excess iron is involved in amyloid beta (Aβ) production and aggregation, causing neuronal cell death [14]. While sustained deviation from redox homeostasis and metal imbalance may promote the development of aged-related neurodegenerative and metabolic diseases, direct evidence for this is limited. Published reports demonstrate that regular exercise can improve iron metabolism and protect against iron accumulation, yet the associated mechanisms remain unclear [15].

Regular exercise has beneficial effects on the whole body and is suggestive of a pathways participating in the interplay between different organs upon exercise training. It has been shown that contracting skeletal muscle produces and secretes different proteins and peptides, so-called myokines, into the bloodstream. The majority of the myokines can cross the blood brain barrier (BBB) and thereby affect brain functions [16]. Interleukin 6 (IL-6) is the first myokine discovered to be released from skeletal muscle in response to physical exercise [17] and is known to evoke systemic anti-inflammatory effects [18]. One session of exercise can increase muscle and plasma IL-6 levels by up to 100-fold, whereas long-term physical training reduces basal IL-6 levels in the plasma [19]. Moreover, given that IL-6 can cross the BBB [18], it is plausible that crosstalk between skeletal muscle and the brain can be mediated by IL-6. Aging and age-related diseases including AD are associated with alterations in myokine levels together with increased chronic inflammation [20]. IL-6, a multifunctional cytokine that is paramount in immune responses and nervous system function, is increased in the brain during aging and AD [21]. In contrast, brain IL-6 levels are reduced upon regular physical exercise [22,23]. Exercise-induced IL-6 modulation remains poorly investigated in the context of AD. Moreover, IL-6 is known to be involved in the regulation of brain iron metabolism [24].

In this study, we used the 5xFAD mouse model of AD, which is an early-onset model with rapid development of a variety of AD-related pathologies, including Aβ plaque deposits accompanied by glial activation starting from 2 months of age, neuronal loss, and cognitive impairments starting from 3–5 months of age [25,26]. The early AD pathology and relatively fast disease development make the 5xFAD mice a suitable model for studying the impact of lifestyle changes, such as physical exercise, in a relatively short period of time, and the model has been widely used in preclinical studies of AD. We have previously shown that, at the age of 7 months, the 5xFAD mice display significant cognitive impairments, which are reversed by long-term voluntary running [27]. While dysregulation of iron homeostasis is evident in AD, little is known about how physical exercise affects iron metabolism in the brain and periphery, and the mechanisms responsible for exercise-induced iron regulation in AD. Therefore, the aim of the present study was to evaluate the effect of long-term voluntary running exercise on iron metabolism in the brain and skeletal muscle in both wild-type (WT) and 5xFAD mice. We also assessed the impact of long-term voluntary running exercise on the regulation of iron metabolism by IL-6.

## 2. Results

**Figure 1:**
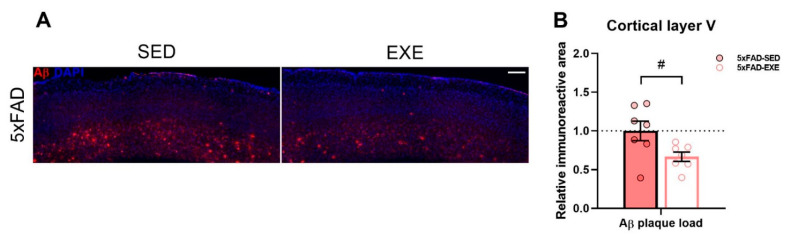
Effects of regular exercise on cortical Aβ load in the 5xFAD mouse model. (**A**) Representative images of Aβ staining in cortical layer V of 5xFAD-SED and 5xFAD-EXE mice. Scale bar 200 μm. (**B**) Percentage of immunoreactive area was quantified to measure Aβ content in cortical layer V. All data are relative to 5xFAD-SED and presented as mean ± SEM. *n* = 8/group. «^#^» exercise effect: ^#^ *p* < 0.05.

5xFAD transgenic male mice and their WT littermates were divided into four groups: WT-sedentary (WT-SED), WT-exercised (WT-EXE), 5xFAD-sedentary (5xFAD-SED), and 5xFAD-exercised (5xFAD-EXE). The long-term voluntary exercise protocol lasted from the age of 1.5 months to 7 months for the exercised mice. No significant differences in running distance were observed among the exercised mice. 5xFAD mice had a lower body mass than the WT mice, and long-term exercise slightly decreased the weights of both WT and 5xFAD mice [27]. To evaluate whether long-term physical exercise affects Aβ plaque load in the brain, we performed immunohistochemical staining using the WO2 antibody of brain sections (Figure 1A). Although physical exercise displayed only a tendency to decrease Aβ plaque load in the hippocampal area of 5xFAD mice [27], in the cortical layer V, the Aβ level was significantly lower in 5xFAD-EXE mice in comparison to 5xFAD-SED mice (*p* < 0.05; Figure 1B).

### 2.1. Exercise Effects on Iron Load in Muscle and Cortex

**Figure 2:**
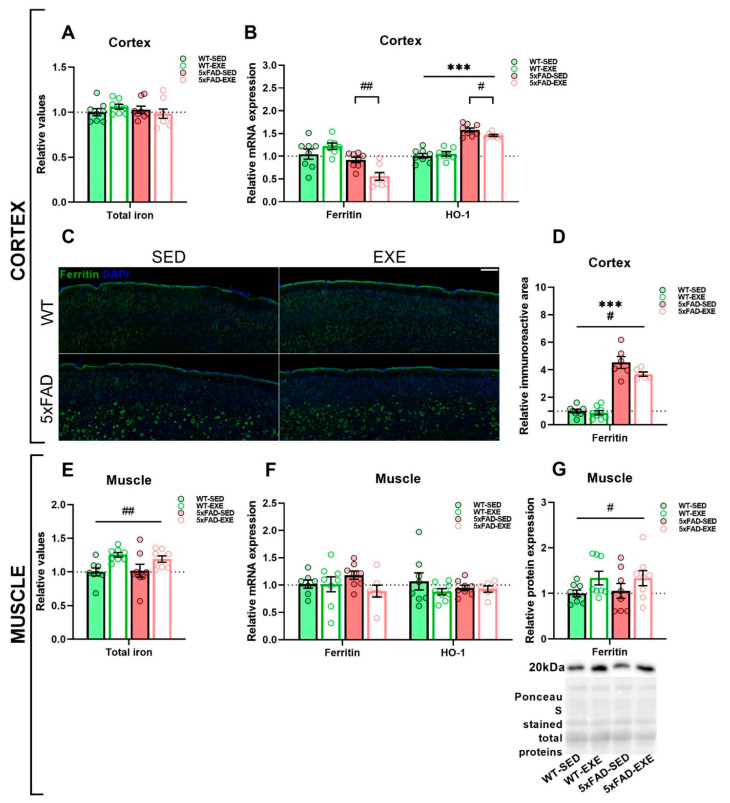
Effects of regular exercise on iron load in cortex and muscle tissues in 5xFAD mouse model. Total iron content, ferritin, and HO-1 level in cortex (**A**–**D**) and muscle samples (**E**–**G**) of WT and 5xFAD mice. Total iron content in cortex (**A**) and muscle (**E**) was measured by ICP-MS. mRNA expression of ferritin and HO-1 in cortex (**B**) and muscle (**F**) was measured by qPCR. (**C**) Representative images of ferritin levels in cortex of WT-SED, WT-EXE, 5xFAD-SED, and 5xFAD-EXE mice. Scale bar 200 μm. (**D**) Percentage of immunoreactive area was quantified to measure ferritin level in cortex. (**G**) Representative Ponceau S staining and Western blot of ferritin in muscle samples and the analysis of band intensities normalized to the total proteins. All data are presented as mean ± SEM. *n* = 8/group. «^#^» exercise effect: *** *p* < 0.001, ^##^ *p* < 0.01, ^#^ *p* < 0.05. General genotype/exercise effect among all groups is presented as a line with «^#^» sign on top, exercise effect in 5xFAD mice only is presented as a bracket with «^#^» sign on top.

Total iron was measured in cortical and gastrocnemius skeletal muscle (muscle) tissues by inductively coupled plasma mass spectrometry (ICP-MS). Although there was no genotype (*p* = 0.6) or exercise effect (*p* = 0.9, Figure 2A) detected in cortical total iron content, a significant exercise-induced increase in total iron level was found in muscles of both WT and 5xFAD mice (main effect of exercise: *p* < 0.01, Figure 2E).

To assess the effect(s) of exercise on iron load, we measured the mRNA expression and protein level of ferritin, the main iron storage protein, in cortex and muscle tissues. Quantitative PCR (qPCR) analysis revealed no changes in mRNA expression of ferritin between WT and 5xFAD mice, neither in cortex (genotype × exercise interaction: *p* < 0.01, post hoc test: *p* = 0.5, Figure 2B) or muscle tissues (main genotype effect: *p* = 0.8, Figure 2F). However, physical exercise induced a significant reduction in the mRNA expression level of ferritin in the cortex of 5xFAD-EXE mice in comparison with 5xFAD-SED mice (genotype × exercise interaction: *p* < 0.01, post hoc test: *p* < 0.01, Figure 2B).

Immunohistochemical staining of brain sections for ferritin (Figure 2C, Supplementary Figure S1A) revealed a significant ferritin increase in the cortex of 5xFAD mice when compared to WT mice (main genotype effect: *p* < 0.001, Figure 2D) while only a slight ferritin increase was detected in the hippocampi of 5xFAD mice when compared to WT mice (main genotype effect: *p* = 0.053, Supplementary Figure S1B). Moreover, physical exercise induced a significant reduction of ferritin in the cortex of exercised mice in comparison to sedentary mice (main exercise effect: *p* < 0.05, Figure 2D), whereas no difference in hippocampal ferritin level was detected between exercised and sedentary mice (main exercise effect: *p* = 0.3, Supplementary Figure S1B). Conversely, Western blot analysis revealed that physical exercise significantly increased the protein level of ferritin in the muscles of exercised mice when compared to sedentary mice (main exercise effect: *p* < 0.05, Figure 2G).

We next assessed heme oxygenase 1 (HO-1), which degrades heme into redox-active Fe^2+^, potentially leading to free iron overload, one of the features of AD [14]. This analysis revealed that HO-1 mRNA expression was significantly upregulated in the cortex of 5xFAD mice (main genotype effect: *p* < 0.0001, Figure 2B). Further post hoc analysis revealed a significant exercise-induced reduction of HO-1 mRNA expression in the cortex of 5xFAD-EXE when compared to 5xFAD-SED mice (*p* < 0.05, Figure 2B). No changes in HO-1 mRNA expression were observed in muscle samples (main genotype effect: *p* = 0.9, main exercise effect: *p* = 0.4, Figure 2F).

### 2.2. Exercise Effects on Iron Trafficking in Muscle and Cortex

**Figure 3:**
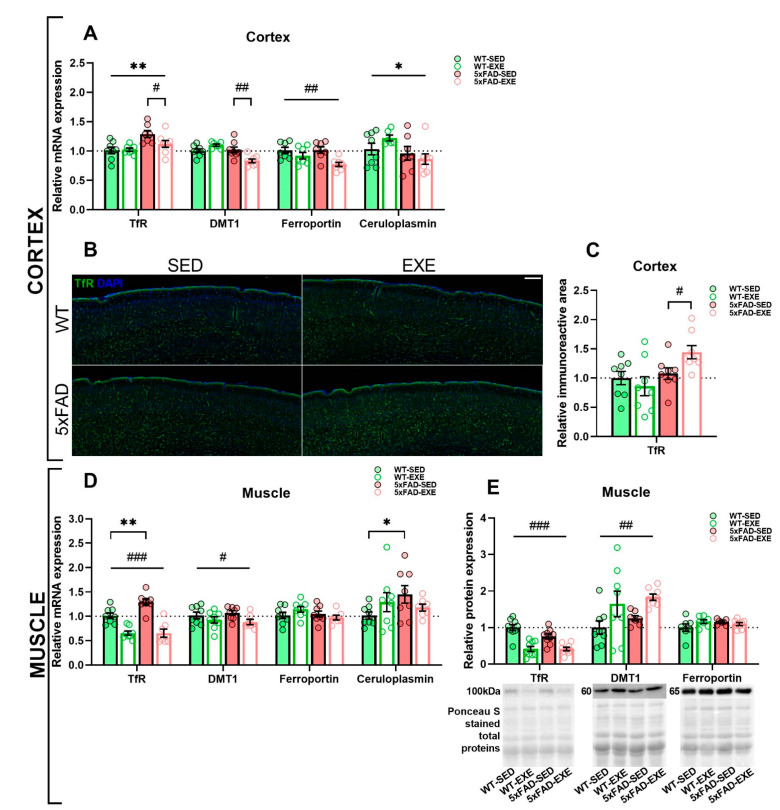
Effects of regular exercise on iron trafficking in cortex and muscle tissues in 5xFAD mouse model. Level of iron transporters and ferroxidase ceruloplasmin in cortex (**A**–**C**) and muscle samples (**D**,**E**) of WT and 5xFAD mice. mRNA expression of TfR, DMT1, ferroportin, and ceruloplasmin in the cortex (**A**) and muscle (**D**) was measured by qPCR. (**B**) Representative images of TfR levels in the cortex of WT-SED, WT-EXE, 5xFAD-SED, and 5xFAD-EXE mice. Scale bar 200 μm. (**C**) Percentage of immunoreactive area was quantified to measure TfR level in cortex. (**E**) Representative Ponceau S staining and Western blot of TfR, DMT1 and ferroportin in muscle samples and the analysis of band intensities normalized to the total proteins. All data are presented as mean ± SEM. *n* = 8/group. «*» genotype effect, «^#^» exercise effect: ** *p* < 0.01, * *p* < 0.05, ^###^ *p* < 0.001, ^##^ *p* < 0.01, ^#^ *p* < 0.05. General genotype/exercise effect among all groups is presented as a line with «*»/«^#^» sign on top, exercise effect in 5xFAD mice only is presented as a bracket with «^#^» sign on top, genotype effect in SED mice only is presented as a bracket with «*» sign on top.

Iron uptake in the brain is regulated by transferrin receptor (TfR) and divalent metal transporter 1 (DMT1) [28]. To investigate the effect of physical exercise on iron uptake, the mRNA and protein level of TfR, which is responsible for Fe^3+^ uptake, were evaluated in cortex and muscle tissues. qPCR analysis demonstrated that the mRNA expression of TfR was upregulated in the cortex (main genotype effect: *p* < 0.01, Figure 3A) of 5xFAD mice and in the muscles of 5xFAD-SED mice when compared to WT-SED mice (genotype × exercise interaction: *p* = 0.07, post hoc test: *p* < 0.01, Figure 3D). Physical exercise induced a significant reduction of cortical TfR mRNA expression in 5xFAD-EXE mice when compared to 5xFAD-SED mice (genotype × exercise interaction: *p* = 0.06, post hoc test: *p* < 0.05, Figure 3A) and in all exercised mouse muscles (main exercise effect: *p* < 0.0001, Figure 3D).

The immunohistochemical staining of brain sections for TfR (Figure 3B) revealed that the protein level of TfR was unchanged in the cortical region of 5xFAD-SED mice compared to WT-SED mice (genotype × exercise interaction: *p* = 0.05, post hoc test: *p* = 0.6, Figure 3C). Physical exercise induced a significant increase of TfR in the cortex of exercised 5xFAD mice in comparison with 5xFAD-SED mice (genotype × exercise interaction: *p* = 0.05, post hoc test: *p* < 0.05, Figure 3C). For muscle tissue, Western blot analysis showed a dramatic decrease of TfR protein in exercised mice when compared to sedentary mice (main exercise effect: *p* < 0.0001, Figure 3E).

In addition, the mRNA expression of DMT1, which transports Fe^2+^ into cells, was analyzed in cortex and muscle tissues by qPCR. Exercise induced a significant reduction in DMT1 mRNA expression in the cortex of 5xFAD-EXE mice in comparison with 5xFAD-SED mice (genotype × exercise interaction: *p* = 0.0004, post hoc test: *p* < 0.01, Figure 3A). Physical exercise caused a similar effect in muscle tissue, slightly reducing DMT1 mRNA expression in all exercised mice in comparison to sedentary mice (main exercise effect: *p* < 0.05, Figure 3D). Conversely, Western blot analysis revealed that regular exercise significantly increased the protein level of DMT1 in the muscles of exercised mice when compared to sedentary mice (main exercise effect: *p* < 0.01, Figure 3E).

To assess the effects of exercise on Fe^2+^ efflux, qPCR analysis was performed to measure mRNA levels of essential iron efflux proteins such as ceruloplasmin and ferroportin. Ceruloplasmin gene expression was reduced in the cortex of 5xFAD mice (main genotype effect: *p* < 0.05, Figure 3A), while increased expression was found in the muscle (genotype × exercise interaction: *p* = 0.1, post hoc test: *p* < 0.05, Figure 3D) in comparison to WT mice. While AD-related changes in ferroportin expression were not detected, physical exercise slightly reduced its expression in the cortex of exercised mice when compared to sedentary mice (main exercise effect: *p* < 0.01, Figure 3A). No significant changes in ferroportin mRNA (Figure 3D) or protein level (Figure 3E) were observed in muscle samples.

### 2.3. Exercise Effects on Regulation of Iron Homeostasis in Muscle and Cortex

**Figure 4:**
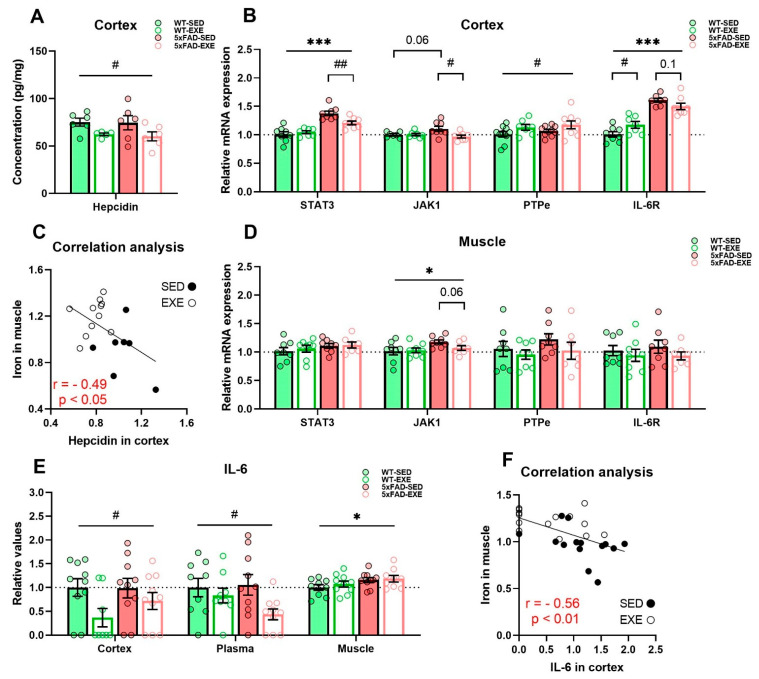
Effects of regular exercise on iron homeostasis regulation in cortex and muscle tissues in 5xFAD mouse model. (**A**) Hepcidin level in cortex was measured by ELISA and normalized to total protein concentration. (**C**) Correlation analysis for total iron content in muscle and hepcidin level in cortex was performed for all mice, r–Pearson correlation coefficient. mRNA expression of STAT3, JAK1, PTPe, and IL-6R in cortex (**B**) and muscle (**D**) was measured by qPCR. (**E**) IL-6 level in cortex, plasma, and muscle protein samples was measured by CBA. (**F**) Correlation analysis for total iron content in muscle and IL-6 level in cortex was performed for all mice. All data are presented as mean ± SEM. *n* = 6–10/group. «*» genotype effect, «^#^» exercise effect: *** *p* < 0.001, * *p* < 0.05, ^##^ *p* < 0.01, ^#^ *p* < 0.05. General genotype/exercise effect among all groups is presented as a line with «*»/«^#^» sign on top, exercise effect in 5xFAD or WT mice separately is presented as a bracket with «^#^» sign/*p* value on top, genotype effect in SED mice only is presented as a bracket with *p* value on top.

Next, we evaluated the effects of exercise on iron homeostasis by measuring the levels of hepcidin, a hormone responsible for regulation of cellular iron levels [29,30]. ELISA analysis revealed a significant reduction of hepcidin in the cortex of exercised mice when compared to sedentary mice (main exercise effect: *p* < 0.05, Figure 4A). Moreover, the signal transducer activator of transcription 3 (STAT3)/Janus kinase 1 (JAK1) pathway, activation of which is known to regulate hepcidin expression [31], was also altered by exercise. qPCR analysis demonstrated significant upregulation of STAT3 mRNA expression (main genotype effect: *p* < 0.001) and a slight increase in JAK1 (*p* = 0.06) in the cortex of 5xFAD mice in comparison with WT, whereas physical exercise ameliorated this increase in STAT3 (genotype × exercise interaction: *p* = 0.02, post hoc test: *p* < 0.01) and JAK1 (genotype × exercise interaction: *p* = 0.03, post hoc test: *p* < 0.05, Figure 4B) in 5xFAD cortices. In addition, an exercise-induced increase in the expression of receptor-type tyrosine-protein phosphatase epsilon (PTPe), which is involved in inhibition of STAT/JAK signaling [32,33], was detected in exercised mice in comparison to sedentary mice (main exercise effect: *p* < 0.05, Figure 4B).

Hepcidin is known to be dependent on circulating iron and inflammation status, particularly on the level of IL-6 [14]. Therefore, we assessed IL-6 receptor (IL-6R) and IL-6 levels in the cortex, plasma, and muscle samples. qPCR analysis demonstrated that the mRNA expression of IL-6R was significantly increased in the 5xFAD cortex when compared to WT (main genotype effect: *p* < 0.001, Figure 4B). Exercise had slight effects in different genotypes: in WT IL-6R, it increased (*p* < 0.05), while in 5xFAD, it tended to decrease (*p* = 0.12, Figure 4B). No differences in IL-6R mRNA expression were detected in muscles (Figure 4D). Cytokine bead array (CBA) analysis revealed that physical exercise induced a significant reduction of IL-6 in the cortex (main exercise effect: *p* < 0.05) and plasma (*p* < 0.05) with no changes in muscle (*p* = 0.5) in both WT and 5xFAD-mice (Figure 4E). In addition, we found a significant negative correlation between cortical IL-6 and iron levels in muscle (r = −0.56, *p* < 0.01, Figure 4F) and cortical hepcidin and iron levels in muscle (r = −0.49, *p* < 0.05, Figure 4C) among all mice: the more iron in muscle, the less IL-6 and hepcidin in the cortex.

## 3. Discussion

Iron is an essential biometal, which is involved in many important biological processes in the body. Heme iron is bound to hemoglobin within red blood cells participating in oxygen transport, whereas non-heme iron is distributed through the body with 10% localized in the brain and participating in neurotransmitter signaling and myelin production [34]. Although AD is associated with iron dyshomeostasis and iron accumulation in the brain [35], the mechanisms underlying iron dysregulation remain unclear. Iron is essential for skeletal muscle oxidative capacity upon exercise, and growing evidence suggests an important role of skeletal muscle and physical exercise for regulation of iron metabolism in the whole body [15,36]. In the present study, we aimed to decipher the role of long-term voluntary running exercise in iron homeostasis in WT mice, and the 5xFAD mouse model of AD. In particular, the aim was to evaluate the effects of exercise on iron load, trafficking, and homeostasis in the brain and skeletal muscles.

Ferritin, the main iron storage protein, is elevated upon aging and in the AD brain. In particular, Aβ plaques found in the AD cortex and hippocampi are associated with iron deposits and ferritin [37,38,39]. In accordance with previous studies of AD model mice [40,41,42], we demonstrated significant increases of ferritin in the cortex of 7-month-old 5xFAD mice. Since ferritin is responsible for attenuation and sequestration of free iron [43], AD-associated increases of ferritin levels may indicate elevated labile iron level in the brain. HO-1 can be partly responsible for elevated labile iron level in the brain, because HO-1 degrades heme into Fe^2+^, leading to labile iron level increase in AD [14]. This notion was supported by our finding of increased HO-1 overexpression in the 5xFAD cortex. Moreover, ferritin upregulation can occur in response to neuroinflammation [44], one of the major pathological features of AD.

Although recent studies demonstrated elevations in total iron levels in the brains of AD mice [41,45], we did not detect increased iron in the cortex of 5xFAD mice by ICP-MS analysis. In the study by Gurel et al., the iron level was measured in hippocampal lysates of 3-month-old 5xFAD mice, an age at which robust Aβ plaque accumulation does not yet occur. In contrast, in our study, the iron level was analyzed at 7 months of age, when Aβ plaque load is three times higher [46]. Iron levels have been shown to depend on age and stage of AD: the iron level has been shown to increase between the ages of 3 and 8 months, with a later iron level decrease occurring until 24 months of age in APP/PS1 mice [40]. Moreover, there is a possibility that AD is associated with changes in the distribution of iron between cell types or between its different molecular forms (free iron, ferritin, transferrin, heme) [14], which may partly explain the unchanged total iron with increased ferritin and HO-1 level in the cortex of 5xFAD mice. Taken together, the findings of our study indicate altered iron regulation via ferritin and HO-1 increases despite no changes in total iron levels in the cortex of 5xFAD mice at 7 months of age.

In recent reviews, it has been suggested that exercise can modulate iron metabolism and reduce iron stores in the body [15,47], but direct evidence is lacking. Furthermore, the effects of physical exercise on iron regulation in the brain remain poorly investigated. In the current study, long-term voluntary exercise reduced ferritin and HO-1 levels coupled with Aβ decrease in the 5xFAD cortex, with no effect on total iron content in the brain. In a recent study, exercise induced a similar reduction of ferritin level in AD mice; however, the total iron content in the brain also decreased [41]. The Choi et al. paper used a different AD mouse model (APP-C105) than that used in the current study and utilized a colorimetric iron measurement technique, which may explain the different results for total iron measurements. While ICP-MS analysis is considered as the golden standard for total iron measurements, colorimetric iron detection may depend on assay conditions, affecting the extent of iron release [48]. While our results demonstrated exercise-induced ferritin and HO-1 reductions in the mouse cortex, ferritin levels and total iron content in muscle were elevated upon exercise with no changes in HO-1 muscular level, which may indicate unchanged labile iron in response to regular exercise in muscle. Mitochondria play an important role in iron metabolism, participating in synthesis of iron sulfur clusters and heme, with the latter molecule being essential for oxygen transport and energy production [49,50]. It is known that exercise training induces increases in skeletal muscle mitochondrial mass, and enhances oxidative muscle capacity and oxygen delivery to skeletal muscle [51]. Thus, increased iron content in skeletal muscle of exercised mice may indicate elevated energy demands in this tissue. Recent work by Ghio et al. demonstrated an increase in total iron content with voluntary exercise in various tissues of rats [52]. In that study, voluntary exercise induced iron redistribution in the body whereby iron level was attenuated in plasma and liver and elevated in tissues with high metabolic activity, such as skeletal muscles, heart and lung. It is therefore plausible that in the current study, regular exercise also induced a redistribution of iron in the body, resulting in ferritin reduction in the cortex and elevation in the muscles coupled with unchanged total iron content in the cortex and elevation in the muscles.

Cellular iron trafficking is regulated by iron transporters including TfR, DMT1, and ferroportin aided by the ferroxidase ceruloplasmin [53]. Given that iron accumulation in the AD brain can be associated with dysregulation of iron trafficking [24], we assessed the effect of exercise on iron transport in the cortex and skeletal muscle tissues. In recent studies, upregulation of iron uptake transporters (TfR and DMT1) and downregulation of iron efflux transporters and responsible proteins (ferroportin and ceruloplasmin) were observed in the brains of various AD mouse models [41,42,54]. In our study, 5xFAD mice had increased TfR and decreased CP in the brain, which is consistent with previous reports. We also found that long-term physical exercise induced a significant reduction of DMT1 together with increased TfR in the 5xFAD brain. To date, only one study evaluated the effect of treadmill exercise on iron transporters in brains of AD model mice, and demonstrated a reduction of TfR and DMT1 with exercise in the motor cortex of APP-C105 mice [41]. The difference in TfR response to exercise may be linked with the use of a different AD mouse model or brain area assessed, or more likely, the exercise regimen used. In our study, we also measured iron transporters in skeletal muscle in response to physical exercise and found dramatic reductions in TfR and increases in DMT1 in the muscles of exercised mice. Similar DMT1 upregulation was observed in skeletal muscle of rats in response to 5-week treadmill exercise [55]. It has been shown that mitochondria express DMT1 transporters [56], which are the major mitochondrial iron importers involved in mitochondrial iron acquisition [57]. We demonstrated increased DMT1 in skeletal muscle in response to exercise, which may indicate increased iron utilization by mitochondria and energy metabolism. Taken together, we found that long-term voluntary exercise induced DMT1 reduction and TfR1 elevation in the 5xFAD cortex, together with DMT1 increase and TfR reduction in skeletal muscles. This may indicate that regularly undertaken exercise is able to modulate iron trafficking both in health and AD. Next, we investigated potential exercise-induced mechanisms responsible for this modulation.

Body iron balance is under the control of hepcidin, a key hormone in iron homeostasis responsible for negative regulation of cellular iron uptake and efflux [24,29,58]. In the periphery, hepcidin is synthetized by the liver and when in circulation, can readily cross the BBB [24]. The synthesis of hepcidin is regulated by iron load and inflammatory status [24]. Previous studies have demonstrated that hepcidin is distributed around Aβ plaques [59], that hepcidin level is increased in the serum of AD patients [60,61,62], and is suggested as a potential blood biomarker for identifying risk of AD [63]. Although we did not observe increased hepcidin in the 5xFAD mice cortex, Wang et al. previously reported elevated hepcidin expression in the mouse brain upon aging [64]. It has been suggested that iron overload occurring in AD is associated with a reduction of iron export due to dysregulation of hepcidin [24]. Thereby a decrease of hepcidin in the brain can potentially have positive effects on stabilization of iron homeostasis in AD [65]. It has been shown that hypoactivity, a model of sedentary behavior, is associated with dysregulation of iron metabolism accompanied by increased hepcidin levels in liver and bone of rats [66,67] and in the spleen and serum of healthy individuals [68]. To date, there are no publications reporting the effect of physical activity on brain hepcidin levels. Here, we report for the first time that long-term voluntary exercise induces a significant reduction of hepcidin in the cortex of both WT and 5xFAD mice. Our study is the first to link the iron status of the brain with reduction of hepcidin in mice undergoing voluntary exercise.

IL-6 is involved in regulation of iron metabolism through hepcidin [24], and IL-6 is necessary for hepcidin induction during inflammation in mice and humans [69]. Moreover, a recent study demonstrated that inflammation-induced iron accumulation and hepcidin upregulation in the brain is regulated by IL-6/STAT3 [31]. In accordance with this report, we observed STAT3 upregulation in the 5xFAD cortex. High levels of IL-6 are associated with cognitive decline and memory impairments [70], and in AD patients, serum and CSF levels of IL-6 are up-regulated and considered as a marker of inflammation [71]. Inflammation mediated by glial cell activation was demonstrated in the 5xFAD brain [27], yet in the current study, we did not observe AD-related increases of IL-6 in the cortex, in line with previously published reports [5,72]. However, we detected a significant increase in IL-6 receptor expression.

While IL-6 is often considered an immune-modulatory cytokine, it is also defined as a myokine secreted from contracting skeletal muscles to the blood stream [16]. Long-term regular aerobic exercise has been shown to reduce basal IL-6 levels in plasma: the more exercise, the lower the basal IL-6 level [73,74,75]. Moreover, IL-6 can cross the BBB, suggesting a potential crosstalk between muscle and the brain [18]. It has been proposed that lowering the peripheral levels of IL-6 may reduce the risk of developing neurocognitive defects [76]. Although regular aerobic exercise causes reductions in serum IL-6 levels in older healthy adults and individuals with mild cognitive impairment [75,77], the effects of exercise on IL-6 level in AD remain poorly studied. Our study demonstrated that long-term voluntary physical exercise significantly reduces IL-6 levels in plasma and the cortex of WT and 5xFAD exercised mice, while IL-6 levels in muscle remain unchanged. Consistent with our findings, previous studies have shown that IL-6 is attenuated in the brain after treadmill exercise of healthy rats [22] and after resistance exercise in APP/PS1 mice [23]. Moreover, a recent human study demonstrated that aging induces increases in serum IL-6, while lifelong aerobic exercise during 50 years decreases serum IL-6 with no differences observed in skeletal muscle IL-6 levels [78]. Together with a reduction of IL-6 in plasma and the cortex, we observed a significant decline of STAT3/JAK1 and upregulation of STAT3/JAK1 inhibitor PTPe in the cortex of exercised 5xFAD mice. We thus propose that long-term voluntary exercise induces a decrease of hepcidin in the brain, possibly via the IL-6/STAT3/JAK1 pathway. The exercise-induced decrease of hepcidin may be central in the regulation of brain iron metabolism.

## 4. Materials and Methods

### 4.1. Experimental Design

This study utilized male 5xFAD transgenic mice carrying five familial AD (FAD)-related mutations in human amyloid precursor protein (APP; Swe, Flo, and Lon) and human presenilin-1 (PSEN1; M146L and L286V) transgenes driven by the mouse Thy1 promoter [42] and their WT littermates on the JAXC57BL/6J background. Starting from six weeks of age, half of the mice were housed in individual regular cages (sedentary, SED), and half were housed in individual cages with a running wheel (Techniplast, Italy) and let to voluntarily exercise (exercise, EXE) freely for 6 months. The running distance and duration were recorded weekly for each exercised mouse using running counters (Sigma, Germany) installed in each cage. Exercised mice were sacrificed two days after removal of the running wheels to avoid acute effects of running. All mice had *ad libitum* access to food and water and were housed under a 12:12-h light-dark cycle with humidity and temperature control. The weights of the mice were monitored on a weekly basis. This study was conducted in accordance with the Council of Europe Legislation and Regulation for Animal Protection and was approved by the National Animal Experiment Board of Finland.

### 4.2. Tissue Collection

At seven months of age, mice were anesthetized with tribromoethanol (Sigma-Aldrich, St. Louis, MO, USA), blood was collected with 3.8% sodium citrate anticoagulant, and mice were transcardially perfused with heparinized saline. The left brain hemispheres were removed, the cortices were dissected on ice and snap frozen in liquid nitrogen and then stored at −70 °C. The right brain hemispheres were fixed for 22 h in 4% paraformaldehyde at +4 °C, followed by 24 h incubation in 30% sucrose at +4 °C, then snap frozen in liquid nitrogen and finally stored at −70 °C for cryosectioning. The right hemispheres (*n* = 8/group) were cut into serial 20 μm sagittal sections, each 400 μm apart, using a cryostat (Leica Microsystems, Wetzlar, Germany) and stored in anti-freeze solution at −20 °C until immunostaining analysis. Gastrocnemius skeletal muscles were collected from perfused animals, snap frozen in liquid nitrogen, and stored at −70 °C until analyzed. Collected blood was centrifuged at 2000× *g* for 6 min at +4 °C, plasma supernatants were additionally centrifuged at 12,000× *g* for 3 min at +4 °C, final plasma samples were snap frozen in liquid nitrogen and stored at −70 °C for further use. 

### 4.3. Iron Quantitation Via ICP-MS

Iron content was assessed in samples via ICP-MS as reported previously [79]. Briefly, mouse brain cortices (*n* = 8/group) were digested overnight in concentrated nitric acid and then heated for 20 min at 90 °C. The volume of each sample was reduced to approximately 40 µL and then diluted to a final volume of 600 µL with 1% (*v/v*) nitric acid diluent. Measurements were performed using an Agilent 7700× series ICP-MS instrument.

Muscle samples were assessed for iron content using “microdroplet” laser ablation-ICP-MS (LA-ICP-MS) as described previously [80]. Briefly, muscle samples (*n* = 8/group) were homogenized in tris(hydroxymethyl)-aminomethane buffered saline (TBS)-based homogenization buffer as described previously [81]. Samples were assessed for protein content using the Pierce BCA Protein Assay kit (Thermo Fisher Scientific, Waltham, MA, USA) and then diluted to a consistent protein concentration. One microliter of each sample was pipetted onto a glass slide and left to air dry overnight. Droplet residues were ablated off the slide surface using laser ablation and analyzed using Iolite software [80]. Measurements were performed using an NWR-213 laser ablation unit (Electro Scientific Industries, Portland, OR, USA) hyphenated to an Agilent 8800 ICP-QQQ-MS.

Iron content was normalized within samples using a multielement control (Mg, P, and K for brain; C and P for muscle) and expressed relative to the WT-SED group.

### 4.4. Protein and RNA Extraction

Cytosolic proteins were isolated from frozen cortical and muscle samples (*n* = 8–10/group) for Western blot, enzyme-linked immunosorbent assay (ELISA), and CBA analysis. Cortical samples were homogenized manually in eight volume of lysis buffer (20 mM Tris, 250 mM sucrose, 0.5 mM EDTA 0.5 mM EGTA, 4% (*v/v*) protease inhibitor cocktail, 1% (*v/v*) phosphatase inhibitor cocktail, pH 7.4) on ice and centrifuged at 5000× *g* for 10 min at +4 °C. Muscle samples were ground into fine powder using a porcelain cup with hammer under liquid nitrogen, then manually homogenized in five volume of lysis buffer (50 mM Tris, 150 mM NaCl, 0.3% Triton X-100, 4% (*v/v*) protease inhibitor cocktail, 1% (*v/v*) phosphatase inhibitor cocktail, pH 7.4) on ice and centrifuged at 1200× *g* for 10 min at +4 °C. The supernatants containing cytosolic proteins were collected and stored at −70 °C for further use. Protein concentrations were determined using the Pierce 660 nm Protein Assay Kit (Thermo Fisher Scientific, Waltham, MA, USA) for cortical samples and using the Pierce BCA Protein Assay Kit (Thermo Fisher Scientific, Waltham, MA, USA) for muscle samples.

Before centrifugation, parts of the cortical and muscle homogenates were taken for RNA isolation, which was performed using TRI reagent (Sigma-Aldrich, St. Louis, MO, USA) according to manufacturer’s instructions. Genomic DNA was removed using DNase I, RNase-free kit (Thermo Fisher Scientific, Waltham, MA, USA) and cDNA was synthesized using High Capacity cDNA Reverse Transcription Kit (Applied Biosystems, Waltham, MA, USA).

### 4.5. Quantitative Real-Time PCR (qPCR)

qPCR analysis was performed to measure mRNA level of essential proteins in iron metabolism in cortical and muscle samples using StepOnePlus Real-Time PCR System (Thermo Fisher Scientific, Waltham, MA, USA). TaqMan gene expression assays (Thermo Fisher Scientific, Waltham, MA, USA) used in this study included Fth1 (ferritin, Mm00850707_g1), Hmox1 (HO-1, Mm00516005_m1), Trfc (TfR, Mm00441941_m1), Slc11a2 (DMT1, Mm00435363_m1), Slc40a1 (ferroportin, Mm01254822_m1), Cp (ceruloplasmin, Mm00432654_m1), Stat3 (STAT3, Mm01219775_m1), Jak1 (JAK1, Mm00600614_m1), Ptpre (PTPe, Mm00448493_m1), Il6ra (IL-6R, Mm00439653_m1), and Gapdh (GAPDH, Mm99999915_g1). Relative expression levels to the WT-SED group were determined using the 2^−ΔΔCt^ method normalized to Gapdh as the endogenous control.

### 4.6. Western Blot

Ferritin and iron transporter protein content in muscles was measured by Western blot. Procedures were performed as previously described [27]. Briefly, 40 μg of muscle proteins were separated by SDS-PAGE, transferred to poly-vinylidene difluoride (PVDF) membranes (GE Healthcare, Chicago, IL, USA), blocked in 5% nonfat dry milk in phosphate-buffered saline with Tween-20 (PBST), washed in PBST, and incubated overnight at +4 °C with primary antibodies against ferritin (1:1000, ab75973, Abcam, Cambridge, UK), TfR (1:1000, ab84036, Abcam, Cambridge, UK), DMT1 (1:1000, ABS983, Sigma-Aldrich, St. Louis, MO, USA), and ferroportin (1:1000, PA5-22993, Invitrogen, Waltham, MA, USA). Then membranes were washed three times in PBST and incubated in goat anti-rabbit secondary antibody (1:3000, conjugated with HRP, 130-65-15, BioRad, Hercules, CA, USA) for 2 h at room temperature. Proteins were visualized with SuperSignalTM West Pico PLUS Chemiluminescent substrate kit (Thermo Fisher Scientific, Waltham, MA, USA), detected with BioRad ChemiDocTM Imaging System, and quantified using ImageLab software (BioRad, Hercules, CA, USA). The results were normalized to the total proteins of Ponceau S staining.

### 4.7. Enzyme-Linked Immunosorbent Assay (ELISA)

The hepcidin concentration was measured in the cortical homogenates using HAMP mouse ELISA kit (Aviva Systems Biology, San Diego, CA, USA) following the manufacturer’s instructions. Absorbance of the samples was read at 450 nm with Wallac Victor 1420 microplate reader (Perkin Elmer, Waltham, MA, USA). All results were normalized by total protein concentration.

### 4.8. Cytokine Bead Array (CBA)

IL-6 concentration was measured in plasma, and cortical and muscle homogenates using the CBA mouse inflammation kit (BD Biosciences, Franklin Lakes, NJ, USA). Samples were run using CytoFlex S flow cytometer (Beckman Coulter, Brea, CA, USA), and acquired data were analyzed with FCAP Array v3.0 software (Soft Flow Ltd., Pcs, Hungary). All results were normalized by total protein concentration and expressed relative to the WT-SED group.

### 4.9. Immunohistochemistry (IHC)

Aβ, ferritin, and TfR levels were evaluated by immunostaining brain cryosections. Three sections (400 μm apart) were washed in 0.1M PB and mounted to superfrost slides (Thermo Fisher Scientific, Waltham, MA, USA). For Aβ and ferritin staining, sections were boiled at +95 °C in 10mM sodium citrate buffer and washed three times in PBST. After blocking in 10% normal goat serum in PBST for 1 h, sections were incubated overnight at room temperature with primary antibodies against Aβ (clone WO-2, 1:1000, MABN10, Sigma-Aldrich, St. Louis, NO, USA), ferritin (1:200, ab75973, Abcam, Cambridge, UK), and TfR (1:200, ab84036, Abcam, Cambridge, UK). Sections were washed three times in PBST and incubated in fluorescent goat secondary antibody (anti-mouse 1:500, AlexaFluor 568, a11004, anti-rabbit 1:250, AlexaFluor 488, a11008; Thermo Fisher Scientific, Waltham, MA, USA) for 2 h. Then, sections were washed three times in PBST and mounted in Vectashield mounting medium with DAPI (H-1200, Vector Laboratories, Burlingame, CA, USA). Images from sections were captured at 10 × magnification by Leica Thunder Imager 3D tissue Slide scanner (Leica Microsystems, Wetzlar, Germany) and analyzed using ImageJ software (National Institute of Health, Bathesda, MD, USA). The percentage of immunoreactive area (positive staining) in the cortex and hippocampus was measured for each section, and the average from three sections per mouse was calculated. All results were expressed relative to the 5xFAD-SED group for Aβ staining, and for WT-SED group for ferritin and TfR staining.

### 4.10. Statistical Analysis

To estimate genotype and exercise difference between WT and 5xFAD mice, two-way analysis of variance (ANOVA) was used. In case of significant interaction between two factors (genotype × exercise), unpaired t test was performed as a post hoc test to examine exercise effect in WT and 5xFAD mice separately or genotype effect in SED and EXE groups separately. Grubbs’ test was performed to determine and remove possible statistical outliers from the analysis. Pearson’s test was used for correlation analysis. All values are expressed as mean ± SEM. Differences were considered significant at *p* < 0.05. Statistical calculations were performed using GraphPad Prism 8.4.2 software (GraphPad Software Inc., San Diego, CA, USA).

## 5. Conclusions

This study highlights the importance of iron dysregulation in AD and demonstrates that long-term voluntary running exercise modulates iron homeostasis in the brain and skeletal muscles of both WT and 5xFAD mice. Our study is the first to link brain alterations of iron homeostasis with decreases in hepcidin and IL-6 in response to regular physical exercise.

## Data Availability

The data presented in this study are available on request from the corresponding author.

## Supplementary Materials

The following are available online at https://www.mdpi.com/article/10.3390/ijms22168715/s1.
