# Supplementary material for: Regular Physical Exercise Modulates Iron Homeostasis in the 5xFAD Mouse Model of Alzheimer’s Disease
Source: Int J Mol Sci. 2021 Aug 13;22(16):8715. doi: 10.3390/ijms22168715 (PMC8395833; doi:10.3390/ijms22168715)
Supplement: Supplementary file 1 [file ijms-22-08715-s001.zip › ijms-1300941-supplementary.pdf]

Supporting information for:

Regular physical exercise modulates iron homeostasis in the 5xFAD mouse model of Alzheimer's disease

Irina Belaya [1], Nina Kucháriková [1], Veronika Górová [1], Kai Kysenius [2], Dominic J Hare [3,4], Peter J Crouch [2], Tarja Malm [1], Mustafa Atalay [5], Anthony R White [6], Jeffrey R Liddell [2], Katja M Kanninen [1,*]

[1] A.I. Virtanen Institute for Molecular Sciences, University of Eastern Finland, 70211, Kuopio, Finland; irina.belaia@uef.fi (I.B.); ninkucha@uef.fi (N.K.); veronika.gorova@uef.fi (V.G.); tarja.malm@uef.fi (T.M.)
[2] Department of Biochemistry and Pharmacology, The University of Melbourne, Melbourne, 3010 Victoria, Australia; kai.kysenius@unimelb.edu.au (K.K.); pjcrouch@unimelb.edu.au (P.J.C.); jliddell@unimelb.edu.au (J.R.L.)
[3] School of BioSciences, The University of Melbourne, Melbourne, 3010 Victoria, Australia; dominic.hare@uts.edu.au (D.J.H.)
[4] Atomic Medicine Initiative, University of Technology Sydney, Broadway, 2007 New South Wales, Australia
[5] Institute of Biomedicine, University of Eastern Finland, 70211 Kuopio, Finland; mustafa.atalay@uef.fi (M.A.)
[6] Mental Health Program, QIMR Berghofer Medical Research Institute, Herston, 4006 Queensland, Australia; tony.white@qimrberghofer.edu.au (A.R.W.)
* Correspondence: katja.kanninen@uef.fi (K.M.K.)

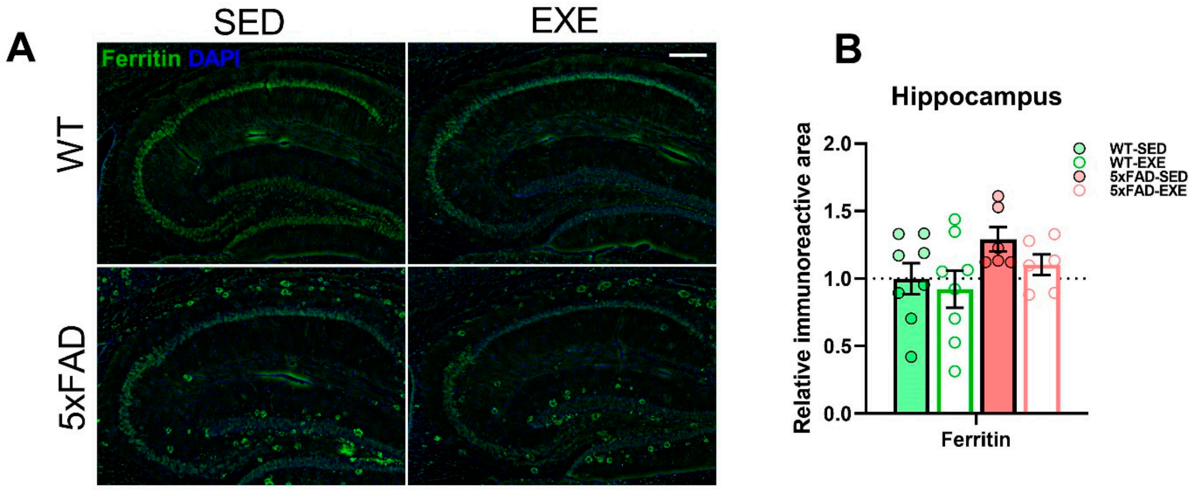

Supplementary Figure S1. Effect of regular exercise on ferritin level in hippocampi. Representative images of Aβ ferritin (**A**) in the hippocampal area of WT-SED, WT-EXE, 5xFAD-SED, 5xFAD-EXE mice. Scale bars: 200 μm. Percentage of immunoreactive area was quantified to measure ferritin (**B**) levels in hippocampi. All data are relative to WT-SED values and presented as mean (±SEM). n=8/group.
